# Thyrotoxic Periodic Paralysis With Severe Hypokalemia Precipitated by Acute Alcohol Intoxication in a Patient With Graves’ Disease

**DOI:** 10.7759/cureus.35548

**Published:** 2023-02-27

**Authors:** Jaskaran Batra, Anvitha Ankireddypalli, Ashok Kumar Kanugula, Swathi Gorle, Jasleen Kaur

**Affiliations:** 1 Department of Internal Medicine, UMPC McKeesport, McKeesport, USA; 2 Department of Endocrinology, University of Minnesota School of Medicine, Minneapolis, USA; 3 Department of Internal Medicine, WellStar Health System-Splading Regional Hospital, Griffin, USA; 4 Department of Internal Medicine, Wellstar Spalding Regional Medical Center, Griffin, USA; 5 Department of Endocrinology, Diabetes and Metabolism, HealthPartners, Minneapolis, USA

**Keywords:** alcohol, periodic paralysis, thyrotoxic, hypokalemia related medical emergencies, hypokalemia

## Abstract

We present a case of a 29-year-old male who presented with thyrotoxic periodic paralysis (TPP) precipitated by acute alcohol intoxication. TPP is an endocrine emergency that presents with an episode of acute flaccid paralysis with hypokalemia in the setting of thyrotoxicosis. Individuals who present with TPP are thought to have an underlying genetic predisposition. Overactivation of the Na^+^/K^+^ ATPase channel leads to large-scale intracellular shifts in potassium, leading to low serum levels and the clinical manifestations of TPP. Severe hypokalemia can lead to life-threatening complications such as ventricular arrhythmias and respiratory failure. Therefore, timely recognition and management are essential in cases of TPP. In addition, understanding the precipitating factors is necessary for adequate counseling of these patients to prevent further episodes.

## Introduction

Thyrotoxic periodic paralysis (TPP) is a rare acquired neuromuscular disorder characterized by episodic weakness or paralysis seen only in a thyrotoxic state of varying severity. A mounting body of evidence suggests that TPP occurs due to a combination of thyrotoxicosis, environmental factors (or precipitating factors), and underlying genetic susceptibility [1]. This condition is most common in the Asian population, followed by the Hispanic population [1]. It remains an unrecognized and underdiagnosed condition in the Western healthcare system [2]. TPP is being increasingly seen in Western countries as they become increasingly diversified due to increased globalization and immigration [3]. The prevalence of TPP is higher in males, despite hyperthyroidism being more prevalent in females [4]. Therefore, early recognition and the institution of appropriate management are essential. We present the case of a young adult with a history of untreated Graves' disease who presented with acute flaccid paralysis and severe hypokalemia precipitated by an episode of acute alcohol intoxication.

## Case presentation

A 29-year male of Southeast Asian descent presented to our emergency department (ED) after sustaining a fall at home. He could not move his extremities when he woke up in the morning. He tried to get out of bed, resulting in a fall and injury to the back of his head. Since morning, he complained of profound muscle weakness, nausea, and vomiting. He mentioned having similar episodes the last year, which lasted for a few hours. He reported drinking 1 liter of vodka (40% alcohol by volume) the previous night. A review of systems was significant for unintentional weight loss of 20-30 pounds over the last 4-6 months, occasional palpitations, and increased anxiety. His medical history is significant for Graves' disease, which was diagnosed a year ago. He was prescribed methimazole but never took his medication. Family history was negative for thyroid disease or similar episodic muscle weakness.

He was afebrile, with a heart rate of 142/minute, blood pressure of 162/85 mm Hg, and respiratory rate of 18/min. His current (BMI) was 26.73 kg/m^2^. On examination, he had mild bilateral exophthalmos. The thyroid gland was non-tender and mildly enlarged with no discreet nodules. The chest was clear to auscultation. Heart sounds were normal with a regular rhythm. His speech was intact, and he was well-oriented. Cranial nerve II-XII assessment was normal. Strength in bilateral upper and lower extremities was 2/5. Grip strength was weak in both hands. Patellar reflexes were hypoactive bilaterally, and sensation to light touch and pinprick was normal.

Laboratory assessment (Table 1) was notable for undetectable serum potassium and mildly low phosphorus level. Complete blood counts and the remaining basic metabolic panel were also normal. The hepatic function panel was also normal. The thyroid workup in Table 1 showed thyrotoxicosis with elevation in thyroid-stimulating hormone (TSH) receptor antibody levels, suggestive of Graves' disease. Troponin T was mildly elevated, suggestive of demand ischemia. His inflammatory markers of erythrocyte sedimentation rate (ESR) and procalcitonin were also mildly elevated. These inflammatory markers were likely elevated due to thyrotoxicosis.

**Table 1:** Laboratory evaluation WBC: white blood cell count; HCO3: bicarbonate; BUN: blood urea nitrogen; TSH: thyroid-stimulating hormone; T4: thyroxine; T3: triiodothyronine; TRAb: TSH receptor antibody; TPO Ab: thyroid peroxidase antibody; BNP: brain natriuretic peptide; AST: asparate transaminase; ALT: alanine transaminase; Alk phos: alkaline phosphatase; ESR: erythrocyte sedimentation rate; CRP: C-reactive protein

Laboratory evaluation	Day 1	Day 1	Day 2	Day 3	Day 4
	Baseline	After 4 hours			
Hemoglobulin (13.3-17.7 g/dL)	15.0				
WBC (4000-11,000 cells/uL)	14,600				
Platelet count (150,000-450,000 cells/uL)	232,000				
Sodium (136-145 mmol/L)	143				
Potassium (3.4-5.3 mmol/L)	<1.5	3.8	4.1	3.7	3.7
HCO3 (22-29 mmol/L)	22				
BUN (6.0-20.0 mg/dL)	18.6				
Creatinine (0.67-1.17 mg/dL)	0.48				
Magnesium (1.7-2.3 mg/dL)	1.7		2.1	1.8	1.9
Phosphorus (2.5-4.5 mg/dL)	2.3		3.9	4.1	4.1
TSH (0.3-4.20 uIU/mL)	<0.01				
Free T4 (0.9-1.70 ng/dL)	7.77		6.03	4.91	4.01
Total T4 (4.5-11.7 ug/dL)	16.8				
Free T3 (2.0-4.4 pg/mL)	22.8		10.2		
Total T3 (55-202 ng/dL)	471				
TRAb (0.0-1.75 IU/L)	33				
TPO Ab (<35 IU/mL)	514				
Cortisol (4-22 ug/dL)	19.6				
Alcohol ethyl (<=0.01 g/dL)	<0.01				
Lipase (13-60 U/L)	27				
Troponin T HS (<=27 ng/L)	47				
N terminal pro-BNP (0-450 pg/mL)	295				
Total bilirubin (<=1.2 mg/dL)	1.0				
AST (10-50 U/L)	36				
ALT (10-50 U/L)	48				
Alk phos (40-129 U/L	124				
Albumin (3.5-5.2 g/dL)	3.9				
ESR (0-15 mm/hr)	18				
CRP (<5.00 mg/L)	4.30				
Procalcitonin (<0.05 ng/mL)	0.11				

**Figure 1:**
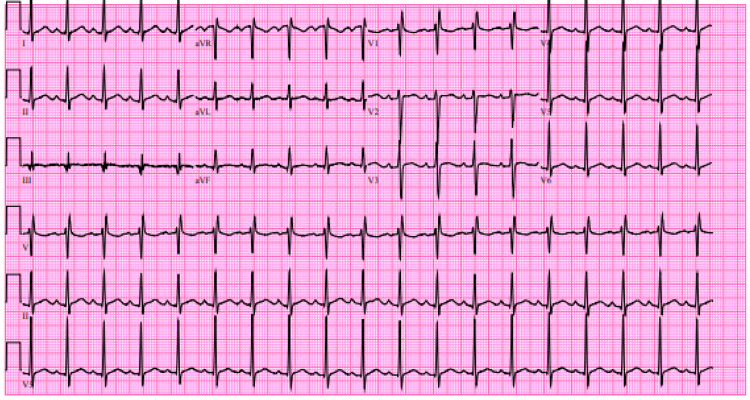
EKG (electrocardiogram) findings.

His urine electrolytes were not evaluated. Electromyograph not performed. Electrocardiogram (EKG) showed sinus tachycardia with non-specific T-wave changes. Atrial and ventricular rate 112/min, PR interval 160 ms (milliseconds), QRS duration 100 ms, and QTc 485 ms. Chest x-ray revealed cardiomegaly with mild vascular congestion. No focal air-space disease or effusions were noted. computed tomography (CT) of the head (without contrast) had no evidence of intracranial hemorrhage, extra-axial collection, mass effect, or acute infarct (Figure 1).

Based on his presentation of acute flaccid paralysis with severe hypokalemia in the setting of untreated Graves' disease, he was diagnosed with TTP. There was also a concern for a thyroid storm due to his presenting symptoms and high thyroid hormone levels (Table 1). His Burch and Wartofsky score was 50. He was given a loading dose of oral propylthiouracil (PTU) 500 mg and then started on 200 mg every six hours. In addition, one dose of intravenous (IV) propranolol 1 mg was given and then started on oral propranolol 40 mg was every six hours. Given the severity of hypokalemia, he was started on potassium replacement therapy. He received 40 mEq of oral potassium chloride (KCl) and started on IV KCl at the rate of 10 mEq/hr (received 30 mEq IV). In addition, he was given IV hydrocortisone 100 mg.

**Figure 2:**
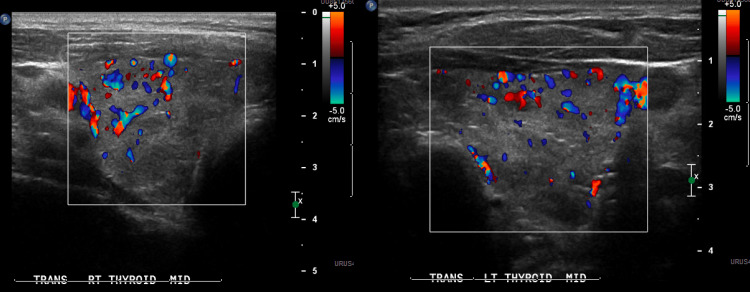
Thyroid ultrasound with color Doppler showing increased blood flow.

After three hours of initiation of therapy, his muscle strength started improving significantly, and he could sit up in bed by himself. Repeat serum potassium in four hours was normal (Table 1), and potassium replacement was discontinued. He underwent further evaluation with a thyroid ultrasound, which showed a diffusely heterogenous thyroid. Increased flow diffusely through the thyroid. Echocardiogram showed normal left ventricular size and function with ejection fraction (EF) of 55%-60%. Mild concentric wall thickening consistent with left ventricular hypertrophy was noted. Left ventricular diastolic function was normal. Assessment of the right ventricle, atria, all valves, and the aortic root was normal. No pericardial effusion was seen (Figure 2).

Genetic testing with genome-wide sequencing was not performed. However, he had a complete recovery of his muscle strength. He was subsequently switched to methimazole 20 mg twice daily and propranolol XL (extended release) 60 mg twice daily and was discharged. He was advised to avoid high carbohydrate intake, alcohol intake, vigorous physical activity, and stress.

## Discussion

Thyrotoxic periodic paralysis is a rare endocrine channelopathy, a complication of thyrotoxicosis seen in patients with an underlying genetic predisposition. Our patient with a history of untreated Graves' disease had presented with acute episodic reversible muscular weakness involving all his extremities and undetectable serum potassium levels. Previous studies have mentioned chronic alcohol use being a risk factor for TPP [5,6]. This is thought to be due to the presence of hypokalemia, a common electrolyte abnormality in patients with chronic alcohol use [5]. In his case, a single episode of excessive alcohol use has likely precipitated TPP. Early recognition and initiation of propranolol therapy with cautious correction of potassium led to rapid resolution of muscular weakness and normalization of serum potassium and phosphate levels.

TPP is more prevalent in Asian populations, with an incidence of 1.8% and 1.9% in Chinese and Japanese populations, respectively [7,8]. In the United States, the incidence reported by the Mayo clinic was much lower, ranging from 0.1% to 0.2% [9]. Despite a higher incidence of hyperthyroidism in females, over 95 percent of thyrotoxic PP cases occur in males, with the male-to-female involvement ratio ranging from 4:1 to 30:1 [1,10]. The typical clinical presentation of TPP would be a younger adult male (rarely female) with a triad of acute muscular weakness/paralysis involving the limbs, hypokalemia, and clinical/biochemical evidence of thyrotoxicosis [1]. These episodes typically occur overnight or early morning hours and seem more prevalent in the summer months [3,4]. They might have experienced similar milder attacks in the preceding months. Severe muscle weakness can rarely result in rhabdomyolysis [11]. Involvement of the respiratory muscles can result in respiratory failure [12]. Involvement of the gastrointestinal muscles can lead to nausea, vomiting, and abdominal distention. There is usually a precipitating event like a high carbohydrate meal, alcohol intake, emotional stress, a high salt diet, rest after strenuous physical activity, smoking, trauma, and menstruation. TPP might be the initial presentation of thyrotoxicosis in up to 50%-70% of the patients who present with this condition [1]. There may be a family history of hyperthyroidism. TPP tends to be sporadic, without any family history of similar episodes, unlike familial hypokalemic periodic paralysis, which has an autosomal dominant pattern and tends to present in the first two decades of life [13]. Biochemical evaluation during the episodes shows varying severity of hypokalemia, mild to moderate hypophosphatemia, normal acid-base balance, and low/suppressed TSH with high-normal/elevated T4 and T3 levels. EKG abnormalities include sinus tachycardia, atrioventricular block, and in extreme cases, ventricular arrhythmias.

Hypokalemia leads to alterations in the generation of action potentials leading to neuromuscular and cardiac manifestations [14]. Hypokalemia seen in patients with TPP is due to the rapid intracellular shift of potassium, leading to low levels in the extracellular space. There is no total body deficit of potassium [3]. This shift is thought to be due to the increased activity of the sodium-potassium adenosine triphosphatase (Na^+^/K^+^ ATPase) pump [1]. Multiple factors, including genetic susceptibility, are believed to be responsible for increased Na+/K+ ATPase activity in TPP^+^/K^+^ ATPase activity in TPP, including genetic susceptibility. The presence is a thyrotoxic state alone is known to stimulate the pump activity. Thyroid hormones regulate beta-adrenergic receptor expression and responsiveness [15]. Beta-adrenergic agonists have been shown to enhance the number and activity of Na^+^/K^+^ ATPases [16]. Thus, thyrotoxicosis further increases Na^+^/K^+^ ATPase pump activity through enhanced beta-adrenergic stimulation. The resolution and prevention of TPP using a non-selective beta-blocker, propranolol, supports this hypothesis [17,18]. Attainment of a euthyroid state also eliminates the possibility of developing TPP.

The genetic pathogenesis of TPP remains to be fully understood. This has been difficult, given the rarity and sporadic nature of the disease. Mutations in the *KCJN18* gene which encodes the Kir2.6 channel, have been reported in 33% of patients with TPP from the United States, Brazil, and France [19]. The Kir2.6 is an inward rectifying potassium channel expressed in skeletal muscles. The thyroid hormone regulates the transcription of this channel [19]. However, this mutation is rarely identified in patients with TPP of Asian descent. Genetic variants in the *KCJN2* gene, which also encodes an inward-rectifying potassium channel, have been associated with increased susceptibility to TPP in Korean and Chinese populations [10,20]. A population-based genome-wide study in the Chinese population identified two new susceptibility foci at *DCHS2* on 4q31.3 and *C11orf67* on 11q14.1 [10]. This study also identified two common risk loci shared by TPP and Graves' disease (MHC and Xq21.1), suggesting that TPP could be a molecular subtype of Graves' disease [10].

Treatment of TPP involves the replacement of potassium, oral or intravenous, at a slow pace to aid in the recovery of muscle strength and avoid serious cardiopulmonary complications. Aggressive potassium replacement should be avoided to prevent rebound hyperkalemia when the episode abates. Replacement should be stopped when recovery of muscle strength is noted. Propranolol should also be added to block the overstimulation of Na^+^/K^+^ ATPase by beta-adrenergic activity. This should be continued until the patient is euthyroid to prevent further TPP episodes. Addressing underlying thyrotoxicosis is the key to the prevention of TPP, usually done with the use of thionamides. Patients should be advised adherence to medication to maintain euthyroidism. They should also avoid precipitating factors like high carbohydrate meals, chronic or occasional excessive alcohol intake, smoking, strenuous physical activity, and significant stress.

## Conclusions

TPP is a rare cause of acute flaccid paralysis seen in patients with thyrotoxicosis, most prevalent in the Asian followed by the Hispanic population. The incidence of this complication is increasing in the Western world due to increased population intermixing. It is essential to identify and manage this condition early due to the potential for infrequent but severe complications like rhabdomyolysis, cardiac arrhythmias, and respiratory failure. The triad of acute limb muscle weakness/paralysis, hypokalemia, and the presence of thyrotoxicosis can be used to identify these patients. Counseling regarding prevention strategies is also critical.

## References

[1] Maciel RM, Lindsey SC, Dias da Silva MR (2011). Novel etiopathophysiological aspects of thyrotoxic periodic paralysis. Nat Rev Endocrinol.

[2] Pompeo A, Nepa A, Maddestra M, Feliziani V, Genovesi N (2007). Thyrotoxic hypokalemic periodic paralysis: an overlooked pathology in western countries. Eur J Intern Med.

[3] Kung AW (2006). Clinical review: Thyrotoxic periodic paralysis: a diagnostic challenge. J Clin Endocrinol Metab.

[4] Lin SH (2005). Thyrotoxic periodic paralysis. Mayo Clin Proc.

[5] Tsai MH, Lin SH, Leu JG, Fang YW (2015). Hypokalemic paralysis complicated by concurrent hyperthyroidism and chronic alcoholism: a case report. Medicine (Baltimore).

[6] Lin YY, Hsieh YS (2021). Chronic alcohol abuse-induced hypokalemia might lead to delayed diagnosis or misdiagnosis of thyrotoxic periodic paralysis. Cureus.

[7] OK S, SH K, II S (1957). The association of periodic paralysis and hyperthyroidism in Japan. J Clin Endocrinol Metab.

[8] Kang MH (2010). 'Kir'-ing thyrotoxic periodic paralysis. Clin Genet.

[9] Kelley DE, Gharib H, Kennedy FP (1989). Thyrotoxic periodic paralysis. Report of 10 cases and review of electromyographic findings. Arch Intern Med.

[10] Zhao SX, Liu W, Liang J (2019). Assessment of molecular subtypes in thyrotoxic periodic paralysis and Graves disease among Chinese Han adults: a population-based genome-wide association study. JAMA Netw Open.

[11] Lee TW, Bae E, Hwang K (2017). Severe hypokalemic paralysis and rhabdomyolysis occurring after binge eating in a young bodybuilder: case report. Medicine (Baltimore).

[12] Liu YC, Tsai WS, Chau T, Lin SH (2004). Acute hypercapnic respiratory failure due to thyrotoxic periodic paralysis. Am J Med Sci.

[13] Statland JM, Fontaine B, Hanna MG (2018). Review of the diagnosis and treatment of periodic paralysis. Muscle Nerve.

[14] Palmer BF, Clegg DJ (2016). Physiology and pathophysiology of potassium homeostasis. Adv Physiol Educ.

[15] Yalcin Y, Carman D, Shao Y, Ismail-Beigi F, Klein I, Ojamaa K (1999). Regulation of Na/K-ATPase gene expression by thyroid hormone and hyperkalemia in the heart. Thyroid.

[16] Bachman ES, Hampton TG, Dhillon H, Amende I, Wang J, Morgan JP, Hollenberg AN (2004). The metabolic and cardiovascular effects of hyperthyroidism are largely independent of beta-adrenergic stimulation. Endocrinology.

[17] Lin SH, Lin YF (2001). Propranolol rapidly reverses paralysis, hypokalemia, and hypophosphatemia in thyrotoxic periodic paralysis. Am J Kidney Dis.

[18] Shayne P, Hart A (1994). Thyrotoxic periodic paralysis terminated with intravenous propranolol. Ann Emerg Med.

[19] Ryan DP, da Silva MR, Soong TW (2010). Mutations in potassium channel Kir2.6 cause susceptibility to thyrotoxic hypokalemic periodic paralysis. Cell.

[20] Park S, Kim TY, Sim S (2017). Association of KCNJ2 genetic variants with susceptibility to thyrotoxic periodic paralysis in patients with Graves’ disease. Exp Clin Endocrinol Diabetes.

